# Coronary vessels contribute to de novo endocardial cells in the endocardium-depleted heart

**DOI:** 10.1038/s41421-022-00486-z

**Published:** 2023-01-10

**Authors:** Mingjun Zhang, Wenjuan Pu, Jie Li, Maoying Han, Ximeng Han, Zhenqian Zhang, Zan Lv, Nicola Smart, Lixin Wang, Bin Zhou

**Affiliations:** 1 State Key Laboratory of Cell Biology, Shanghai Institute of Biochemistry and Cell Biology, Center for Excellence in Molecular Cell Science, University of Chinese Academy of Sciences, Chinese Academy of Sciences, Shanghai, China; 2 School of Life Science and Technology, ShanghaiTech University, Shanghai, China; 3 Department of Physiology, Anatomy and Genetics, British Heart Foundation Centre of Regenerative Medicine, University of Oxford, Oxford, UK; 4 Department of Cardiac Surgery, Zhongshan Hospital, Fudan University, Shanghai, China; 5 Key Laboratory of Systems Health Science of Zhejiang Province, School of Life Science, Hangzhou Institute for Advanced Study, University of Chinese Academy of Sciences, Hangzhou, China

**Keywords:** Transdifferentiation, Transdifferentiation

Dear Editor,

During heart development, coronary vessels are mainly derived from two progenitor populations: sinus venosus (SV), the vein that returns blood to the embryonic heart^1^, and endocardium, the endothelial cell layer that lines the lumen of the heart^1,2^. SV-derived coronary vessels primarily populate the outer ventricular myocardial wall, while endocardium-derived coronary vessels largely populate the inner ventricular myocardial wall of the neonatal heart^3,4^. Endocardial progenitors are able to expand and compensate for the loss of SV-derived vessels^5^. While endocardial-to-endothelial conversion has been intensively studied in recent years, it remains unknown whether coronary vessels can reversibly convert back to endocardial cells. Of note, the endocardium plays a crucial role in cardiac valve formation and normal heart function^6^. Understanding the cellular plasticity that allows committed cells to revert to their parental cells is fundamentally important for cell plasticity research and regenerative medicine applications^7^. We, therefore, explored the capability of coronary vessels to convert to endocardial cells.

To enable efficient lineage tracing of endocardial cells, we generated a new tool, *Npr3-tTA* knock-in mouse line, in which a cDNA encoding tetracycline transactivator (tTA) was driven by endocardial marker Npr3^8^ (Fig. 1a). By crossing *Npr3-tTA* with *TetO-Cre* and *R26-LSL-tdT* (*Rosa26-loxP-Stop-loxP-tdTomato*), we generated *Npr3-tTA;TetO-Cre;R26-LSL-tdT* mice, in which tTA binds the *TetO* sequence and activates expression of the downstream Cre for genetic lineage tracing of Npr3^+^ endocardial cells. Whole-mount fluorescence of postnatal day (P)2 hearts and immunostaining for tdTomato (tdT) and VE-Cad on P2 heart sections revealed tdT^+^ endothelial cells within left atria (LA), right atria (RA), ventricular septum (VS) and inner ventricular wall (Fig. 1b, c), confirming the successful generation of a TetO system for genetic targeting of the endocardium.

**Fig. 1:**
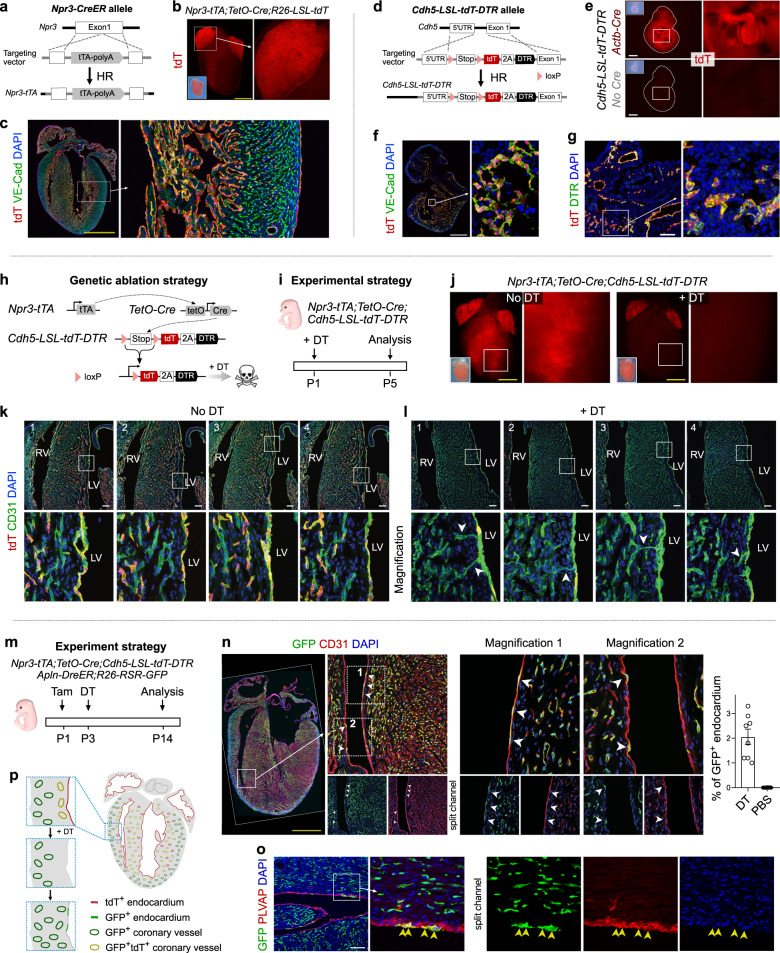
Coronary endothelial cells contribute to endocardial cells after genetic depletion of endocardium. **a** Schematic showing knock-in strategy for generating *Npr3-tTA* allele. **b** Whole-mount fluorescence image of P2 heart. **c** Immunostaining for tdT and VE-Cad on heart sections from (**b**). **d** Schematic showing knock-in strategy for generation of *Cdh5-LSL-tdT-DTR* allele. **e** Whole-mount fluorescence image of E10.5 embryos. **f**, **g** Immunostaining for tdT and VE-Cad (**f**) or DTR (**g**) on E10.5 *Cdh5-tdT-DTR;Actb-Cre* embryonic sections. **h** Schematic showing genetic ablation of endocardium-derived endothelial cells. **i** Schematic showing the experimental strategy. **j** Whole-mount fluorescence image of P5 heart from *Npr3-tTA;TetO-Cre;Cdh5-LSL-tdT-DTR* mice treated with or without DT. **k**, **l** Immunostaining for tdT and CD31 on P5 heart sections treated without DT (**k**) or with DT (**l**). Arrowheads indicate connection between coronary vessels and the endocardium. **m** Schematic showing the experimental strategy for simultaneous coronary vessel tracing and endocardial cell depletion. **n** Immunostaining for GFP and CD31 on P14 heart sections treated with DT. Arrowheads, GFP^+^CD31^+^ cells. Right panel shows the quantification of the percentage of GFP^+^ endocardium. Data are means ± SEM; *n* = 8. **o** Immunostaining for GFP and PLVAP on P14 heart sections treated with DT. Arrowheads, GFP^+^PLVAP^+^ cells. **p** Cartoon image showing coronary endothelial cells convert to endocardial progenitors after depletion of endocardium-derived cells. Scale bar 1 mm (yellow), 100 µm (white).

Previous studies documented the conversion of daughter cells to parental progenitors by exploiting the diphtheria toxin receptor (DTR) to ablate progenitors^7^. To evaluate the potential for coronary vessels to convert to endocardial progenitors, we used *Npr3-tTA;TetO-Cre* to genetically ablate endocardial cells and assess whether coronary vessels can generate de novo endocardium. Since endocardial cells contribute many cell types other than vascular endothelial cells (VECs)^9^ and Npr3 is additionally expressed in epicardial cells^8^, we used the intersection of *Npr3* and pan-endothelial cell *Cdh5* expression to specifically ablate the endocardium-derived endothelial cell compartment. To do this, we generated a Cre-*loxP* activated, endothelial cell-specific, inducible DTR mouse line *Cdh5-LSL-tdT-DTR* (Fig. 1d). The Cre-dependent expression of tdT and DTR in VE-Cad^+^ cells indicated the successful generation of an endothelial cell-specific DTR tool (Fig. 1e–g).

By crossing *Npr3-tTA;TetO-Cre* with *Cdh5-LSL-tdT-DTR*, we generated a compound line in which tTA-activated Cre-*loxP* recombination drives tdT and DTR expression specifically in Npr3^+^ endothelial cells of the endocardium and coronary vessels (Fig. 1h). Immunostaining for tdT, CD31, and endocardial cell marker PLVAP on P7 *Npr3-tTA;TetO-Cre;Cdh5-LSL-tdT-DTR* heart sections showed tdT^+^ endothelial cells in the LA, RA, VS and inner myocardial wall (Supplementary Fig. S1b). To determine the efficiency of tdT^+^ cell ablation, we treated neonatal mice with diphtheria toxin (DT) and found a substantial portion of tdT^+^ endothelial cells were eliminated after 3 days. After 5 days, almost all tdT^+^ cells were eliminated and the endocardium started to be reconstructed and, by 12 days, both coronary vessels and endocardium were large repaired (Supplementary Fig. S1a, c). TUNEL assay showed DT treatment induced robust apoptosis compared with no DT treatment (Supplementary Fig. S1d). Quantification of the percentage of tdT^+^ endocardial cells revealed no significant regional variations in the ablation efficiency and repairing process among VS, RV, and LV (Supplementary Fig. S2).

We next explored the process of endocardial reconstruction. 5 days after DT treatment (Fig. 1i), whole-mount fluorescence imaging showed a near-complete depletion of tdT^+^ signal in the ventricles (Fig. 1j). Immunostaining for tdT and CD31 on serial heart sections showed that some coronary vessels connected to the endocardium in DT-treated hearts (Fig. 1l), whereas no such connections were detected in the absence of DT treatment (Fig. 1k). To examine if coronary vessels contribute endocardial cells in the setting of cell ablation, we used *Apln-DreER* to trace coronary vessels, as *Apln* is robustly expressed in coronary vessels, but not in endocardium^10^. We generated an *Apln-DreER;R26-RSR-GFP;Npr3-tTA;TetO-Cre;Cdh5-LSL-tdT-DTR* mouse to simultaneously deplete the endocardial cell lineage and genetically trace coronary vessels. We treated pups with tamoxifen at P1 and DT at P3, and collected hearts for analysis at P14 (Fig. 1m). Immunostaining showed some GFP^+^CD31^+^ endocardial cells after DT treatment, and quantification data showed GFP^+^ endocardium constituted 2.06% ± 0.31% of the ventricular endocardium in DT-treated mice (Fig. 1n). Immunostaining for GFP and PLVAP confirmed that the GFP^+^ cells in the innermost layer were indeed endocardial cells (Fig. 1o). These results suggested that coronary vessels could convert to endocardium after endocardial cell depletion (Fig. 1p). To delineate the transformation process in more details, we treated mice with a lower dose of DT, following tamoxifen treatment at P1, and analyzed P7 and P14 hearts (Supplementary Fig. S3a). Immunostaining showed some GFP^+^ coronary channels connected with the endocardium at P7 and some GFP^+^PLVAP^+^ endocardial cells which were folded within the myocardium and segregated from the chamber at P14 (Supplementary Fig. S3b–d). We also observed some GFP^+^ cells expressing lower PLVAP levels abutting PLVAP^+^ cells along the endocardial lining (Supplementary Fig. S3e), suggesting a dynamic ongoing conversion of VEC to endocardial cells for endocardial reconstruction after damage (Supplementary Fig. S3f). We did not detect any GFP^+^PLVAP^+^ endocardial cells in the *Apln-DreER;R26-RSR-GFP;Npr3-tTA;TetO-Cre;Cdh5-LSL-tdT-DTR* mouse without DT injection, indicating no vessel-to-endocardium conversion under normal conditions (Supplementary Fig. S4a, b). The absence of GFP^+^ cells on heart sections from *R26-RSR-GFP;Npr3-tTA;TetO-Cre;Cdh5-LSL-tdT-DTR* mouse with DT (Supplementary Fig. S4c, d) excluded any potential Cre-*rox* recombination in our system. Of note, coronary endothelial cells in the adult heart still keep the capability of generating new endocardial cells after genetic depletion of the endocardium (Supplementary Figs. S5–S6).

In this work, we generated two novel mouse lines, *Npr3-tTA* and C*dh5-LSL-tdT-DTR*, to achieve the efficient and specific elimination of endocardial cells and their coronary vessel descendants, but not other cell lineages. To assess the potential of surviving coronary vessels in reconstituting endocardium, we used a dual genetic system employing the Dre-*rox* system to fate map coronary vessels simultaneously with endocardial cell ablation. These results demonstrated that survived coronary vessels could expand to compensate for the loss of coronary vessels derived from the endocardium. Since most coronary vessels from the endocardium were ablated, most of the survived coronary vessels are likely derived originally from SV. Furthermore, the labeled coronary vessels migrated towards and connected with the endocardium, and adopted endocardial cell fate by expressing some specific markers for endocardium. Our study not only shows that coronary vessels from SV could compensate for the loss of endocardium-derived coronary vessels but further reveals a high degree of cell fate plasticity within coronary vessels that could be exploited to generate de novo endocardial cells. Despite long-established progenitor-derivative cell hierarchy, we show that the boundary between these discrete endothelial cell types can be overridden upon activation of inducible gene programs. The molecular mechanisms underpinning these conversions would be worthy of future investigation.

## Supplementary information

Supplementary Information
