# Author Correction: Coronary vessels contribute to de novo endocardial cells in the endocardium-depleted heart

**DOI:** 10.1038/s41421-023-00524-4

**Published:** 2023-03-03

**Authors:** Mingjun Zhang, Wenjuan Pu, Jie Li, Maoying Han, Ximeng Han, Zhenqian Zhang, Zan Lv, Nicola Smart, Lixin Wang, Bin Zhou

**Affiliations:** 1 State Key Laboratory of Cell Biology, Shanghai Institute of Biochemistry and Cell Biology, Center for Excellence in Molecular Cell Science, University of Chinese Academy of Sciences, Chinese Academy of Sciences, Shanghai, China; 2 School of Life Science and Technology, ShanghaiTech University, Shanghai, China; 3 Department of Physiology, Anatomy and Genetics, British Heart Foundation Centre of Regenerative Medicine, University of Oxford, Oxford, UK; 4 Department of Vascular Surgery, Zhongshan Hospital (Xiamen), Fudan University, Xiamen, Fujian, China; 5 Department of Vascular Surgery, Zhongshan Hospital, Fudan University, Shanghai, China; 6 Key Laboratory of Systems Health Science of Zhejiang Province, School of Life Science, Hangzhou Institute for Advanced Study, University of Chinese Academy of Sciences, Hangzhou, China

**Keywords:** Transdifferentiation, Transdifferentiation

Correction to: *Cell Discovery* (2023) 9:410.1038/s41421-022-00486-z published online 10 January 2023

In this article, the authors have found an error about one author’s affiliation. The affiliation for Prof. Lixin Wang should be: Department of Vascular Surgery, Zhongshan Hospital (Xiamen), Fudan University, Xiamen, Fujian, China.

We are sorry for the inconvenience.

