# Supplementary material for: Coronary vessels contribute to de novo endocardial cells in the endocardium-depleted heart
Source: Cell Discov. 2023 Jan 10;9:4. doi: 10.1038/s41421-022-00486-z (PMC9832008; doi:10.1038/s41421-022-00486-z)
Supplement: Supplementary file 1 — Supplementary Information [file 41421_2022_486_MOESM1_ESM.pdf]

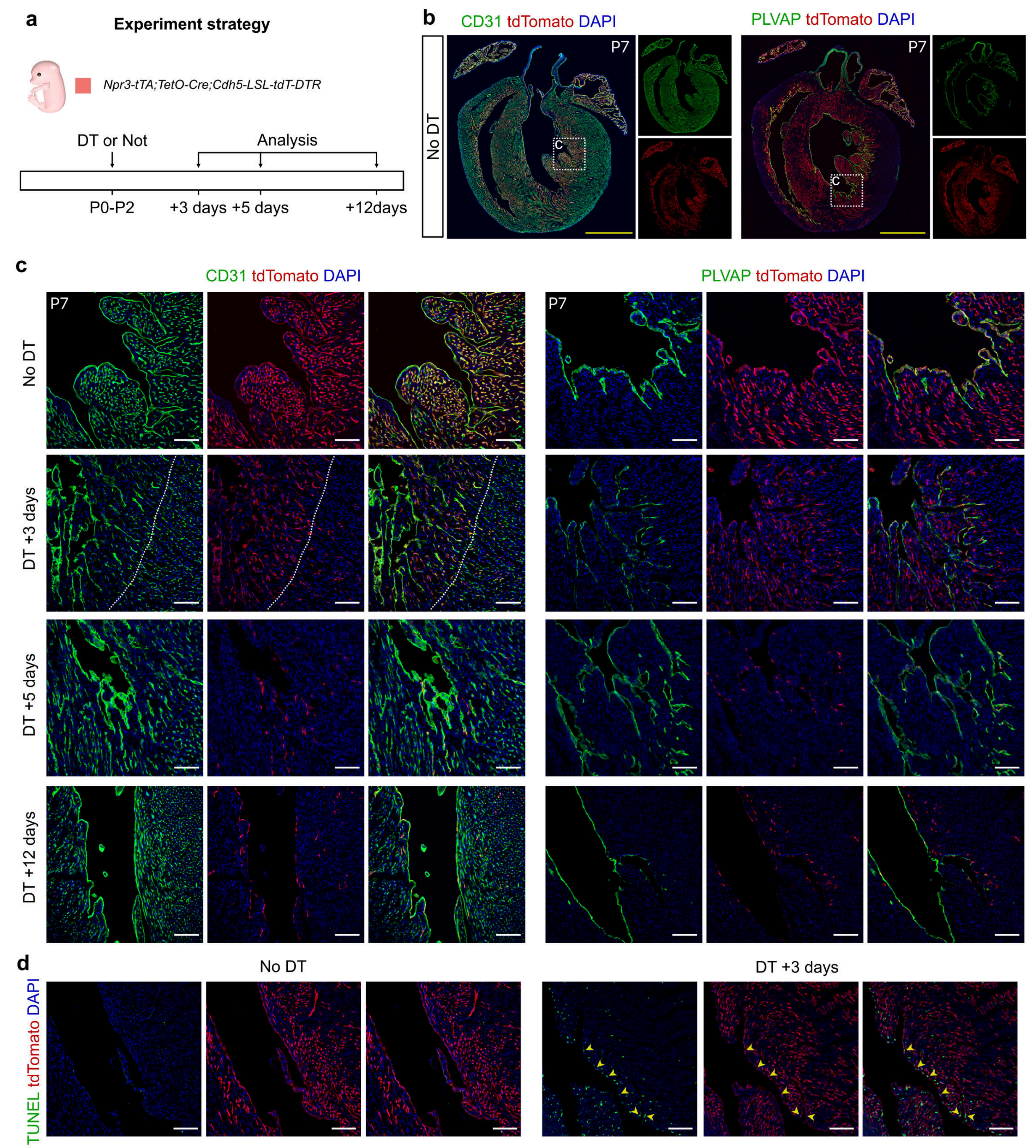


**Supplementary Fig. S1 DT treatment clear the endocardium. a** Schematic figure showing the experiment strategy. **b,c** Immunostaining for tdTomato, PLVAP, and CD31 on heart section from indicated *Npr3-tTA;TetO-Cre;Cdh5-LSL-tdT-DTR* mice. **d** Immunostaining for tdTomato and TUNEL on heart section from indicated *Npr3-tTA;TetO-Cre;Cdh5-LSL-tdT-DTR* mice. Yellow arrowhead, TUNEL⁺ endocardium. Scale bar: white, 100μm. Each figure is representative of 5 individual biological samples.

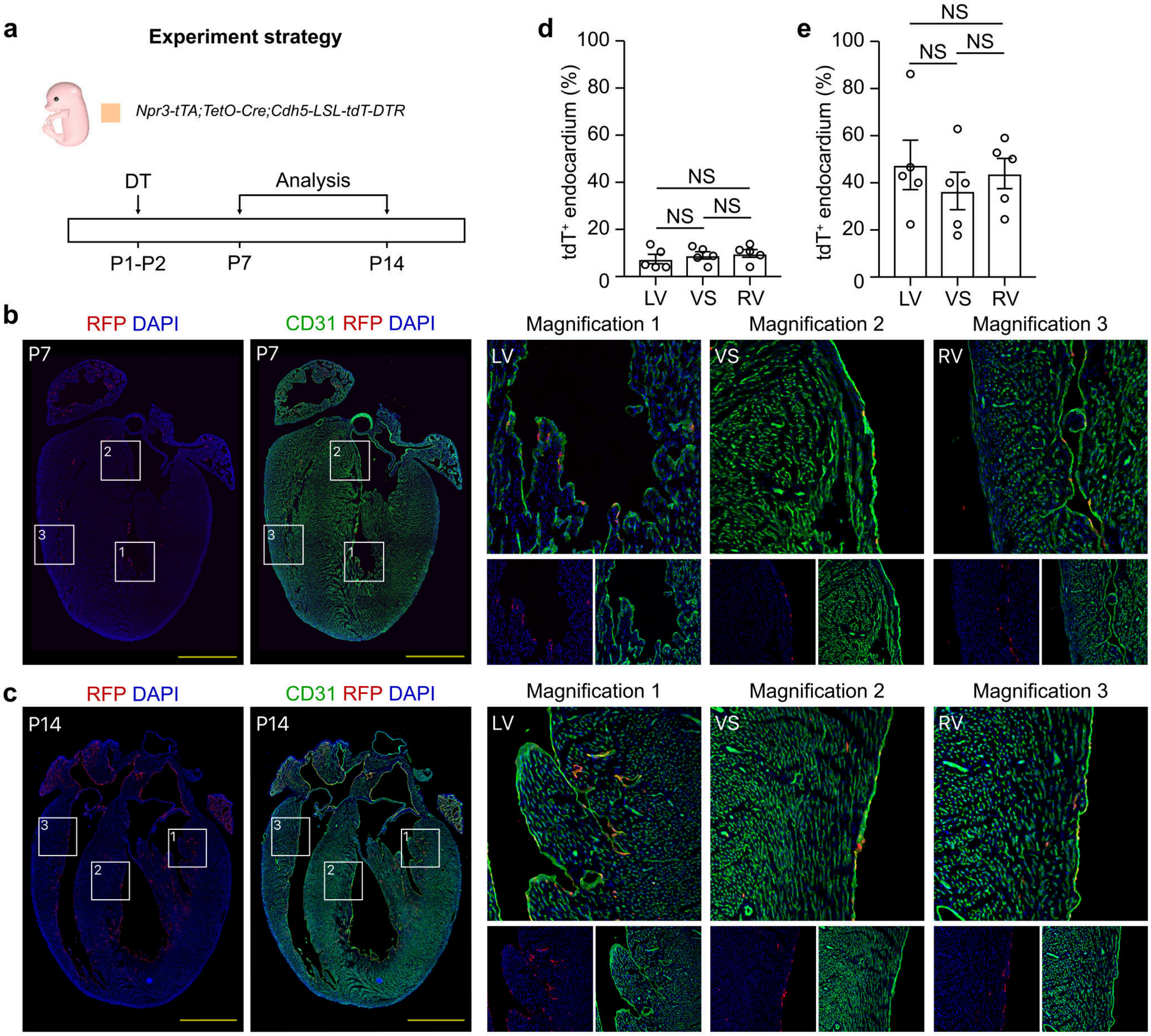


**Supplementary Fig. S2 Clearance of endocardial cells in different regions of heart. a** Schematic figure showing the experiment strategy. **b,c** Immunostaining for tdTomato and CD31 on heart section from P7 (**b**) or P14 (**c**) *Npr3-tTA;TetO-Cre;Cdh5-LSL-tdT-DTR* mice. Scale bar: yellow, 1mm. **d,e** Quantification of the percentage of tdTomato⁺ endocardium in different region 5 day (d) and 10 days (e) after DT treatment. Data are mean ± SEM; n=5. LV, left ventricle; VS ventricle septum; RV, right ventricle.

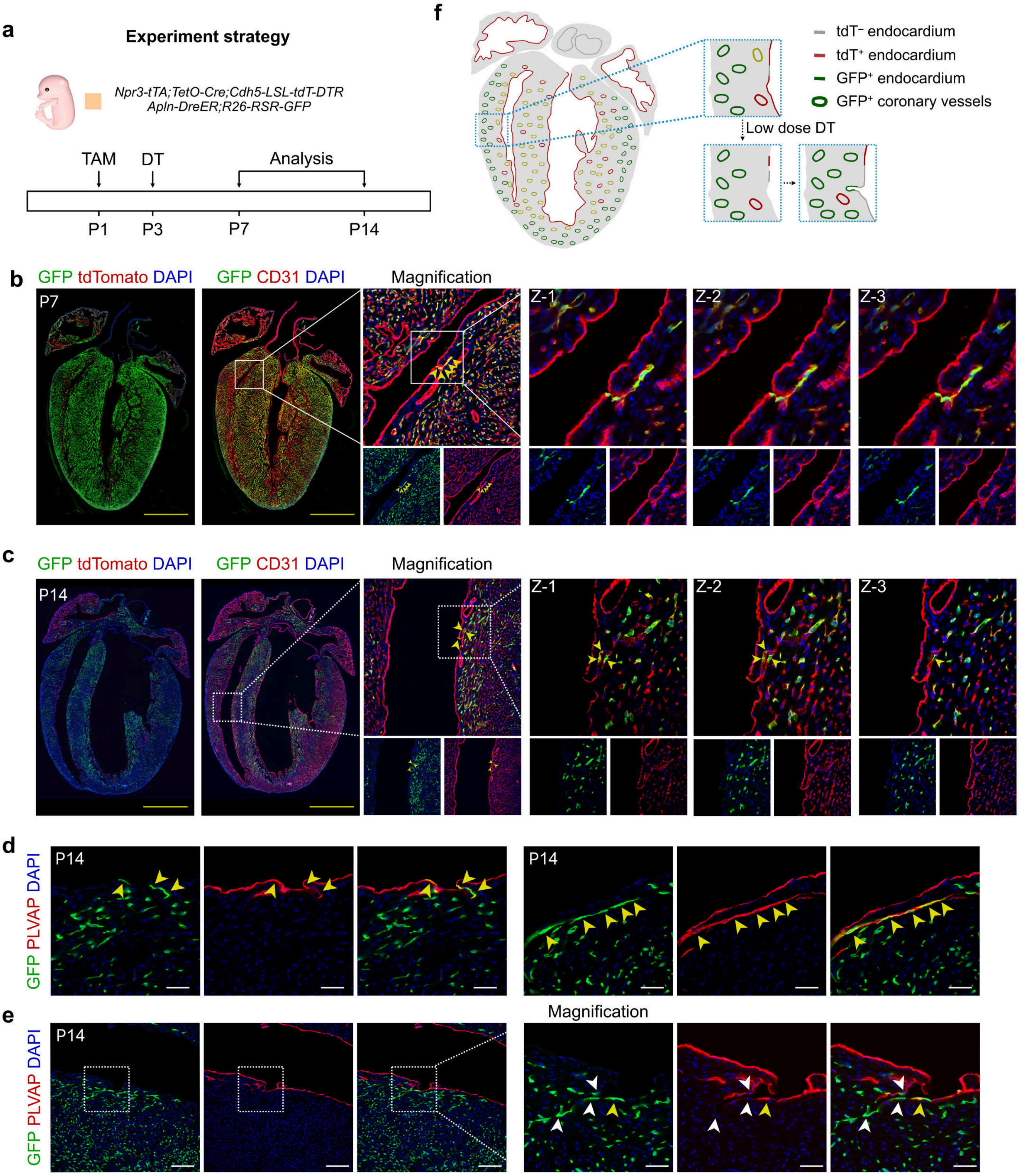


**Supplementary Fig. S3 Coronary vessels connect to endocardium and differentiate into endocardium. a** Schematic figure showing the experiment strategy. **b** Immunostaining for tdTomato, GFP, and CD31 on heart section from P7 *Apln-DreER;R26-RSR-GFP;Npr3-tTA;TetO-Cre;Cdh5-LSL-tdT-DTR* mice. **c** Immunostaining for tdTomato, GFP, and CD31 on heart section from P14 *Apln-DreER;R26-RSR-GFP;Npr3-tTA;TetO-Cre;Cdh5-LSL-tdT-DTR* mice. **d,e** Immunostaining for GFP and PLVAP on heart section from indicated P14 *Apln-DreER;R26-RSR-GFP;Npr3-tTA;TetO-Cre;Cdh5-LSL-tdT-DTR* mice. Yellow arrowheads, $GFP^{+}PLVAP^{+}$ cells; white arrowheads, $GFP^{+}PLVAP^{-}$ or $GFP^{+}PLVAP^{low}$ cells connected with endocardium. Scale bar: white, 100μm. **f** Cartoon figure shows the potential of coronary vessels to generate endocardial cells. Each figure is representative of 5 individual biological samples.

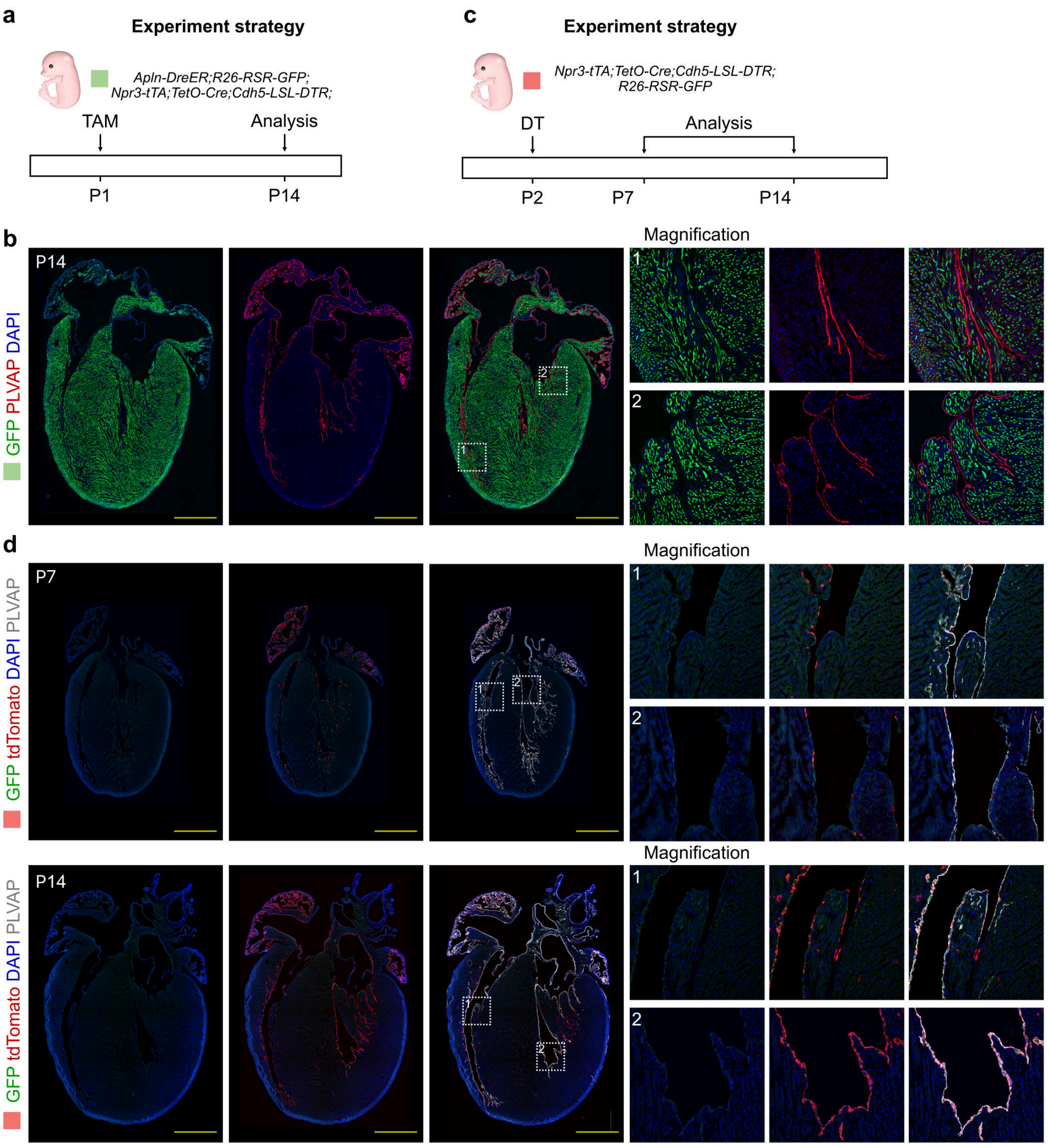


**Supplementary Fig. S4 No Dre-*loxp* recombination in *Npr3-tTA;TetO-cre;Rosa26-rsr-GFP* mice and no ectopic endocardial labeling by *Apln-DreER*. a,c** Schematic figure showing the experiment strategy. **b** Immunostaining for GFP and PLVAP on heart section from P7 *Apln-DreER;R26-RSR-GFP;Npr3-tTA;TetO-Cre;Cdh5-LSL-tdT-DTR* mice. **d** Immunostaining for GFP, RFP, and PLVAP on heart section from P7 and P14 *Npr3-tTA;TetO-Cre;Cdh5-LSL-tdT-DTR;R26-RSR-GFP* mice. Scale bar: yellow, 1mm. Each figure is representative of 5 individual biological samples.

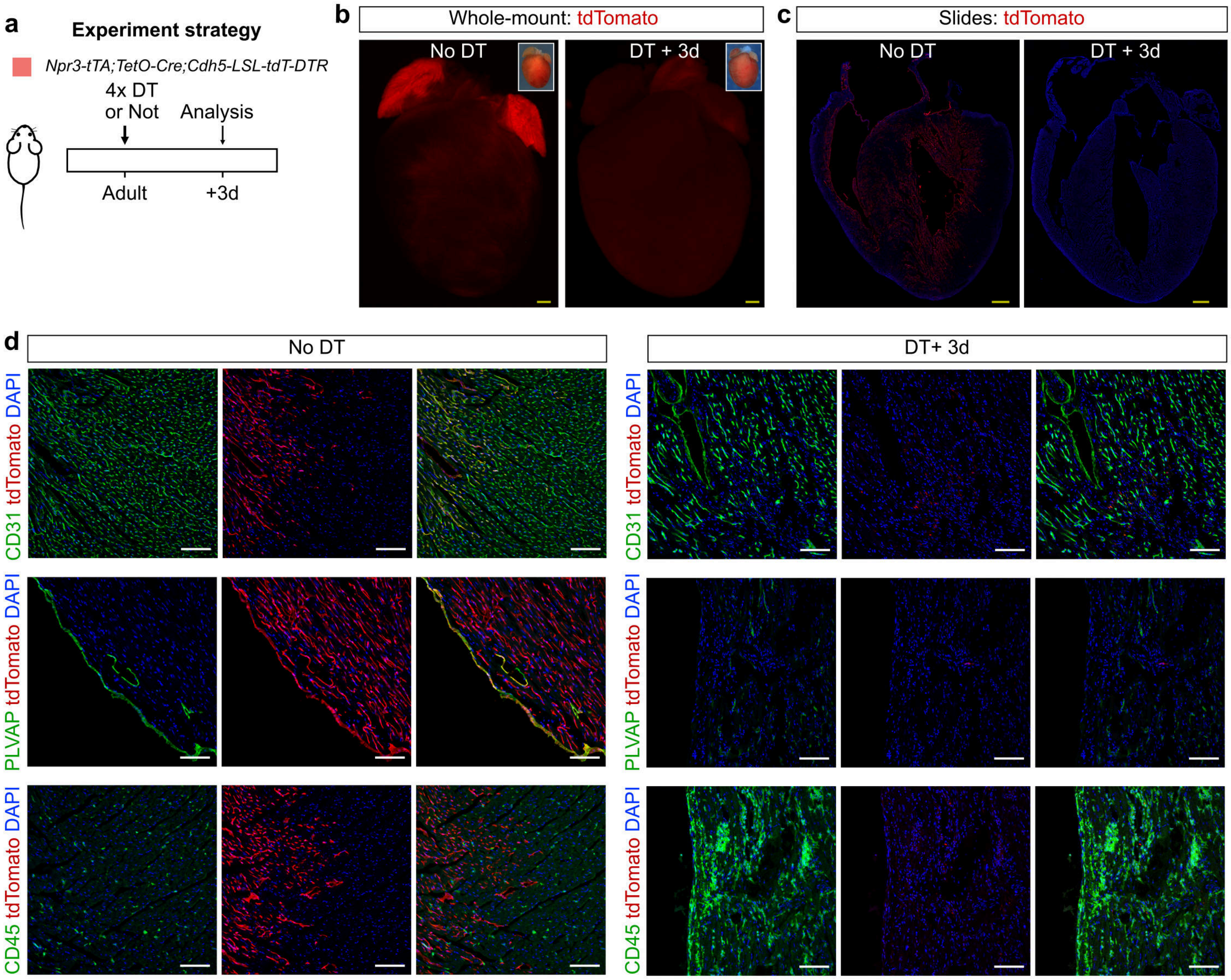


**Supplementary Fig. S5 Ablation of adult endocardium by DT treatment. a** Schematic figure showing the experiment strategy. **b** Whole-mount fluorescence image of adult heart from *Npr3-tTA;TetO-Cre;Cdh5-LSL-tdT-DTR* mice treated without DT or with DT. **c** Immunostaining for tdTomato on adult heart section from *Npr3-tTA;TetO-Cre;Cdh5-LSL-tdT-DTR* mice treated without DT or with DT. **d** Immunostaing for CD31, PLVAP, CD45, and tdTomato on adult heart section from *Npr3-tTA;TetO-Cre;Cdh5-LSL-tdT-DTR* mice treated without DT or with DT. Scale bar: yellow, 1mm; white, 100μm. Each figure is representative of 5 individual biological samples.

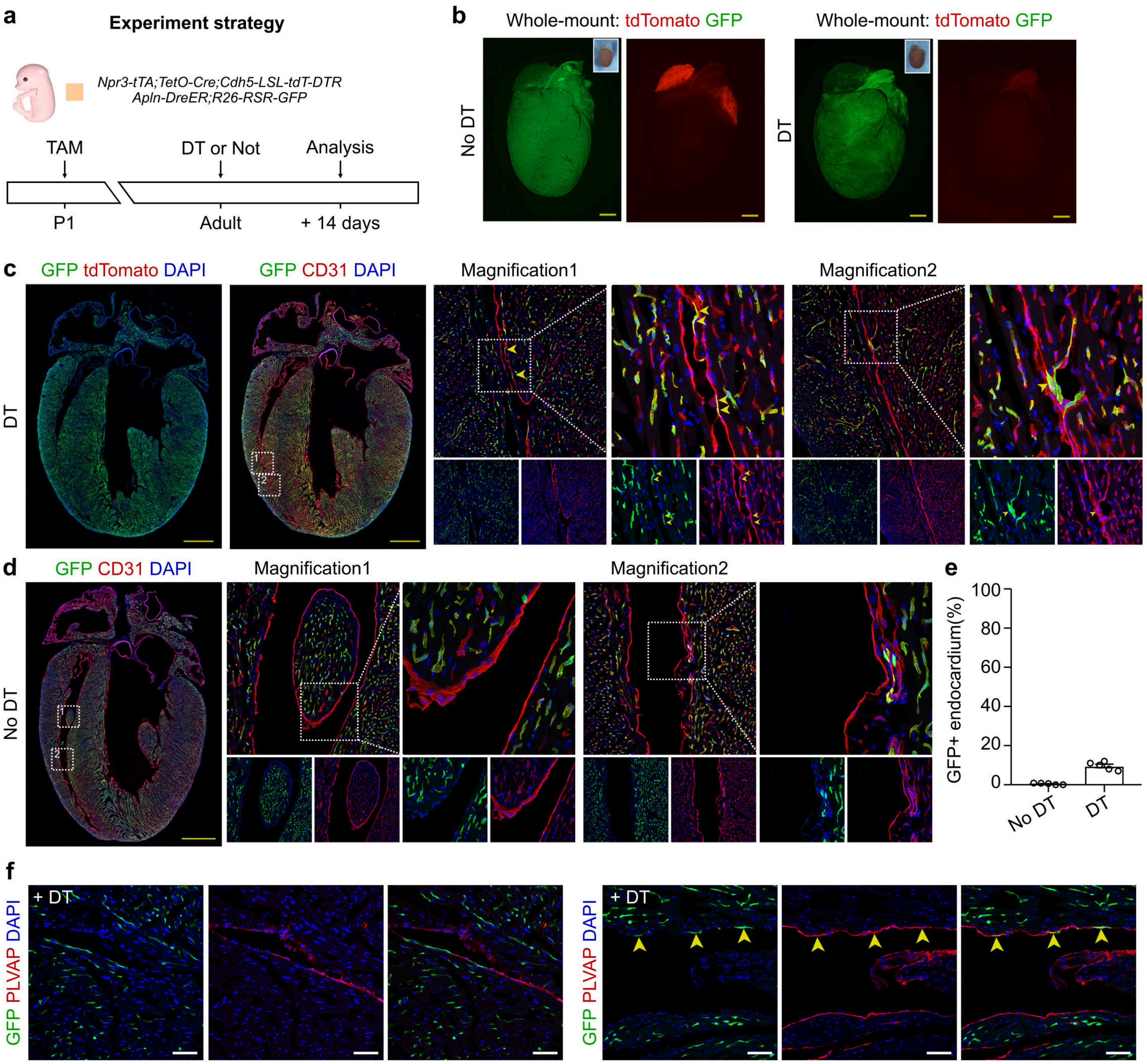


**Supplementary Fig. S6 Coronary vessels could contribute to endocardium in adult heart. a** Schematic figure showing the experiment strategy. **b** Whole-mount fluorescence image of adult heart from *Apln-DreER;R26-RSR-GFP;Npr3-tTA;TetO-Cre;Cdh5-LSL-tdT-DTR* mice treated without DT or with DT. **c,d** Immunostaining for tdTomato, GFP, and CD31 on heart section from adult *Apln-DreER;R26-RSR-GFP;Npr3-tTA;TetO-Cre;Cdh5-LSL-tdT-DTR* mice treated with DT or not. Yellow arrowheads, $GFP^{+}CD31^{+}$ cells. **e** Quantification of the percentage of coronary vessels-derived endocardium. Data are mean ± SEM; n=5. **f** Immunostaining for GFP and PLVAP on heart section from adult *Apln-DreER;R26-RSR-GFP;Npr3-tTA;TetO-Cre;Cdh5-LSL-tdT-DTR* mice treated with DT. Yellow arrowheads, $GFP^{+}PLVAP^{+}$ cells. Scale bar: yellow, 1mm; white, 100μm. Each figure is representative of 5 individual biological samples.
